# Physicochemical Properties of Freeze–Dried Bigel-Based Materials Composed of Sodium Alginate/Whey Protein Isolate Hydrogel and Ethylcellulose/Sunflower Oil Oleogel

**DOI:** 10.1021/acs.biomac.4c01677

**Published:** 2025-03-25

**Authors:** Weronika Walendziak, Timothy E. L. Douglas, Justyna Kozlowska

**Affiliations:** † Faculty of Chemistry, Nicolaus Copernicus University in Torun, Gagarina 7, 87-100 Torun, Poland; ‡ School of Engineering, Lancaster University, Gillow Avenue, Lancaster LA1 4YW, U.K.

## Abstract

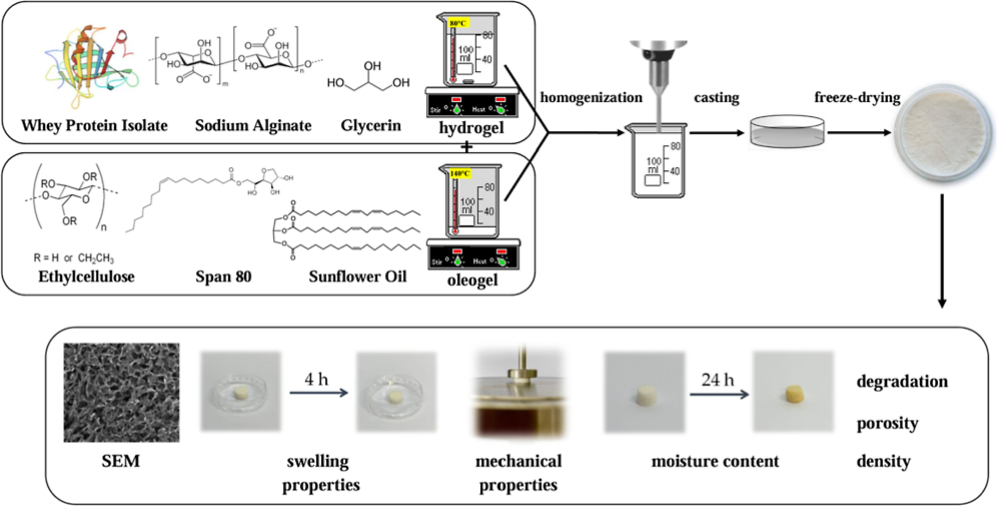

Freeze–drying bigels is a novel technique for developing functional materials for dermatological and cosmetic use, leveraging the benefits of two structured phases. This study optimized freeze–dried bigels composed of whey protein isolate (WPI)/sodium alginate/glycerin hydrogel and ethylcellulose (EC)/Span 80/sunflower oil oleogel at varying hydrogel/oleogel ratios. The materials showed swelling ratios from 50% to 255%, with higher values for a lower oleogel content and higher polymer concentration. The higher oleogel content extended the degradation from a few hours to 7 days. The polymer concentrations and hydrogel/oleogel ratios influenced Young’s modulus (1.25–3.7 MPa). Porosity varied from 35% to 58%, and density varied from 100 to 200 mg/mL. The residual moisture content (5% to 20%) increased with EC content and decreased with WPI and oleogel content. These findings underscore the role of polymer concentrations and phase ratios in tuning the physicochemical properties of freeze–dried gels, positioning them as promising biomaterials for skincare and cosmetic applications.

## 1 Introduction

Hydrogel is a three-dimensional network of hydrophilic polymer chains that can hold large amounts of water. Hydrogels are materials widely investigated for wound healing, cosmetic, biomedical, pharmaceutical, drug delivery, and tissue engineering applications.^1,2^ Whey protein isolate (WPI) and sodium alginate can be classified as relatively cheap and versatile polymers, creating hydrogel networks. WPI is a byproduct of the dairy industry, obtained during the industrial production of cheese from bovine milk. Caseins and whey protein constitute the main proteins in ruminant milk. Lipids, carbohydrates, and lactose are removed during the purification process, resulting in a product containing at least 90% proteins. The main components of WPI are β-lactoglobulin and α-lactalbumin; however, glycomacropeptide, immunoglobulins, bovine serum albumin, lactoferrin, lysozyme, prosthetic peptones, and others are also present.^3^ Sodium alginate is a linear block copolymer composed of α-l-guluronic and β-d-mannuronic acid residues connected by a glycosidic bond. Homogeneous blocks composed only of the residue of one or the other acids are separated by blocks of random or alternating units of α-l-guluronic and β-d-mannuronic acids.^4^ This naturally occurring anionic polysaccharide is obtained from the cell walls of marine algae, mainly brown algae (*Phaeophyceae*).^5^

Oleogel is usually prepared by gelation of polymeric organogelators or through self-assembly and noncovalent bonds with low-molecular-weight organogelators, such as fatty acids, fatty alcohols, waxes, lecithin, cyclodextrins, and others.^6^ Ethylcellulose (EC) may be employed as a polymeric organogelator for different oils due to its ability to structure liquid oil directly.^7^ It is a cellulose derivative, a polysaccharide linear polymer that goes through a glass transition at around 140 °C. The structure of EC-based oleogel significantly depends on the fatty acid profile of oil.^8^ Saturated oil tends to create a softer gel, whereas with the increase of unsaturation, the hardness of the gel also increases. This is due to the more efficient packing of unsaturated lipids into the EC network, resulting in the increase in the oil density and hence promoting the gel strength.^9,10^ Several oils have already been reported to be used in the preparation of oleogels, with sunflower oil among them.^11^ Sunflower oil primarily comprises linoleic acid, a polyunsaturated fat, and oleic acid, a monounsaturated fat.^12^ The oil also contains a large amount of tocopherols. Therefore, it is a suitable oil to select in order to prepare materials intended for the skin.

Bigels are semisolid systems formed by combining a hydrogel, based on hydrophilic polymers, and an oleogel—oil gelled with an organogelator. These two immiscible gels are mixed at a high shear rate and specific temperature. There are several parameters crucial to the physicochemical properties of bigel-based materials, such as the composition of aqueous and oily phases gelled with suitable polymers (hydrophilic for hydrogels and lipophilic or amphiphilic for oleogels), the concentration of gelling agents, as well as mixing proportion of oleogel and hydrogel.^13,14^ The main mechanism behind the bigels formation is the physical interactions between the two structured phases, including hydrogen bonding, which has been found to play an important role in the structure of bigels.^15,16^

Bigels’ many advantages over other semisolid formulations resulting from combining two structured phases have drawn recent scientific attention, mainly concerning food applications^17−20^ and topical administration of drugs and active substances.^21−23^ These systems present better physicochemical properties and stability than single gel.^24,25^ Moreover, they enable the delivery of both hydrophilic and lipophilic ingredients individually and simultaneously, as well as the control of their release due to blending both structured phases.^26^ Nonoily nature, easy spreadability to the skin, and enhancement of the *stratum corneum* hydration are their further assets.^27^ The tailorable properties of bigels, owing to the modification of each gel composition and their combinations, make them suitable materials for various applications.

Modification of bigels by freeze–drying may lead to further enhancement of their characteristics for cosmetic and dermatological applications. Freeze–drying is a dehydration process that involves removing the solvent via sublimation of frozen samples at a reduced temperature and under reduced pressure. This leads to obtaining almost anhydrous, light, and porous materials with a three-dimensional structure. Research on sodium alginate and WPI-based hydrogels has shown that freeze–drying leads to optimal porosity and water absorption for effective wound healing and drug delivery.^28,29^ Hydrogels based on sodium alginate have been shown to enhance moisture retention and biodegradability, while WPI improves mechanical strength and bioactive compound encapsulation.^30,31^ Similarly, studies on oleogel-hydrogel systems suggest that freeze–drying improves the oil binding capacity and mechanical strength of materials.^32,33^ Hydrophilic–lipophilic balance of bigels is crucial for maintaining their stability,^34,35^ and freeze–drying may disrupt this balance, leading to phase separation. To address this, incorporating stabilizers or cryoprotectants could help maintain the structural integrity of freeze–dried bigels.^36^ Cryopreservation is expected to enhance porosity and water absorption due to the prevention of ice crystal formation while also improving elasticity by preventing excessive protein aggregation and maintaining a more flexible, stable structure.^37^ To the best of our knowledge, there are few reports of freeze–dried bigel-based materials, mainly for medical uses, revealing significant changes in their physicochemical properties.^38,39^ However, the effect of freeze–drying on bigels remains underexplored, highlighting the need for further investigation. Moreover, the idea of using such materials for cosmetic purposes is an innovation in cosmetic chemistry.

This study explores the impact of freeze–drying on bigels, addressing a gap in current research where most studies focus on bigels, oleogels, or freeze–dried hydrogels. By modifying the structure through freeze–drying, we aim to enhance porosity, swelling behavior, mechanical strength, and degradation control, making these materials more suitable for dermatological and transdermal drug delivery applications. These findings will support the development of next-generation bigel-based formulations with optimized functional properties, which will contribute to pharmaceutical, biomedical, and cosmetic applications, offering stable, effective, and scalable solutions for advanced skincare and medical treatments.

This research aimed to optimize the methodology for obtaining materials based on freeze–dried gels and characterize these materials. The hydrogel comprised sodium alginate, WPI, and glycerin, whereas the oleogel was composed of sunflower oil, EC, and Span 80. They were blended at different hydrogel/oleogel ratios using a homogenizer, frozen, and freeze–dried. Subsequently, they were characterized by scanning electron microscopy (SEM), degradation properties, mechanical properties, moisture content, swelling properties, porosity, and density. Freeze–drying of bigels exhibits an unprecedented approach to formulating modern functional materials that may be implemented in dermatological and cosmetic applications.

## 2 Materials and Methods

### 2.1 Materials

WPI (BiPRO, Davisco Foods International Inc., Eden Prairie, MN) with 97.7% protein and 75% β-lactoglobulin in DM (according to the manufacturer’s specification) was used. Sodium alginate (ALG) was obtained from BÜCHI Labortechnik AG (Flawil, Switzerland) with the viscosity average molecular weight equal to 55,800 for *K* = 0.0178 cm^3^/g and *a* = 1.^40^ EC and Span 80 were acquired from Sigma-Aldrich (Poznan, Poland). Glycerin, sodium phosphate, and disodium phosphate were purchased from Chempur (Piekary Slaskie, Poland). Isopropanol was supplied from Stanlab (Lublin, Poland). Sunflower oil was obtained from Nanga (Zlotow, Poland). All chemicals used were of analytical grade.

### 2.2 Materials Preparation

**Figure 1:**
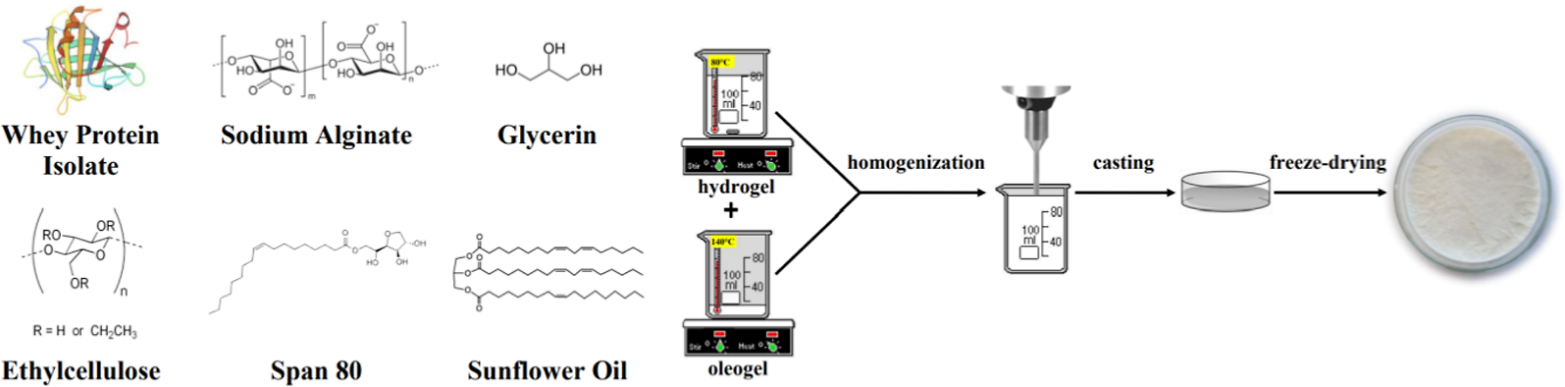
Freeze–dried bigel-based materials preparation method.

Bigels were obtained by mixing hydrogel (containing 1% or 3% of WPI, 2% of ALG, and 1% of glycerin dissolved in water) and oleogel (composed of 10% or 15% of EC and 1% of Span 80 dissolved in sunflower oil) in three oleogel/hydrogel mixing ratios, 5/95, 10/90, and 15/85 (Table 1). Both phases were heated on the magnetic stirrer at a speed of 400 rpm until suitable temperatures of both gels were obtained: hydrogel to 70–80 °C, whereas oleogel to 140 °C which is a temperature of EC dissolution (Figure 1). Afterward, they were mixed and homogenized (20,000 rpm, 3 min) (T25 digital ULTRA-TURRAX disperser, IKA Werke, Staufen, Germany). The solutions were cast on glass plates that were subsequently frozen (−20 °C) and freeze–dried (−55 °C, 5 Pa, 24 h) (ALPHA 1–2 LD plus lyophilizator, Martin Christ, Osterode am Harz, Germany).

**Table 1:** Compositions of Prepared Freeze–Dried Bigels^a^

sample	ratio hydrogel/oleogel	composition of materials (%, w/w)				
		hydrogel			oleogel	
		WPI	ALG	G	EC	Span 80
10% EC + 1% WPI + 2% ALG	5/95	1	2	1	10	1
	10/90	1	2	1	10	1
	15/85	1	2	1	10	1
10% EC + 3% WPI + 2% ALG	5/95	3	2	1	10	1
	10/90	3	2	1	10	1
	15/85	3	2	1	10	1
15% EC + 1% WPI + 2% ALG	5/95	1	2	1	15	1
	10/90	1	2	1	15	1
	15/85	1	2	1	15	1
15% EC + 3% WPI + 2% ALG	5/95	3	2	1	15	1
	10/90	3	2	1	15	1
	15/85	3	2	1	15	1

a WPI—whey protein isolate; ALG—sodium alginate; G—glycerin; and EC—ethylcellulose.

### 2.3 Materials Characterization

#### 2.3.1 Imaging

Structures and cross sections of obtained porous materials were evaluated by SEM imaging (Quanta 3D FEG scanning electron microscope, Quorum Technologies, Lewes, UK). Before the analysis, the surfaces of the materials were covered with thin layers of gold and palladium (SC7620 mini Sputter Coater/Glow Discharge System, Quorum Technologies, Lewels, UK).

#### 2.3.2 Swelling Properties

In order to establish the swelling properties of 3D samples, weighed dry samples (*W*_d_) were immersed in buffer saline (PBS, pH 5.7) for 4 h. After that time, samples were removed from PBS solution and weighted (*W*_w_). Measurements were carried out in triplicate. The swelling ratio (1) was expressed as the percentage ratio of increased weight to the initial weight, as follows
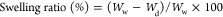
1

#### 2.3.3 Degradation Properties

Degradation of bigel-based materials were conducted by determining percentage weight loss of samples incubated in PBS (pH = 5.7) at room temperature. Weighed samples (*W*_b_) were put in 24-well polystyrene plates and immersed in PSB for 6, 12, 18 h, 1, 2, 3, 5, and 7 days. After each incubation time, samples were removed from the PBS and rinsed with deionized water three times. Frozen samples (−20°) were lyophilized (−55 °C, 5 Pa, 24 h) (ALPHA 1–2 LD plus freeze-dryer, Martin Christ, Osterode am Harz, Germany) and weighed again (*W*_a_). The percentage weight loss was carried out in triplicate and calculated according to eq 2
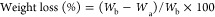
2

#### 2.3.4 Mechanical Properties

Mechanical properties of freeze–dried bigels were established using a mechanical testing machine (Shimadzu EZ-Test EZ-SX, Kyoto, Japan) fitted with a 50 N load cell. Cylindrical samples with a 10 mm diameter were compressed at a 5 mm/min compression speed. Stress–strain curves were recorded using the Trapezium X Texture program (version 1.4.5.), from which Young’s modulus, compressive strength, and yield strength were calculated as the average values of seven measurements.

#### 2.3.5 Porosity and Density Measurements

The porosity (*Ε*) and the density (*d*) of the obtained 3D materials were evaluated by a liquid displacement method using isopropanol as nonsolvent of used polymers: WPI, sodium alginate, and EC.^41^ Moreover, isopropyl alcohol is able to easily permeate through the matrices and not cause swelling or shrinkage. Weighed samples (*W*) were placed in the graduated cylinder previously filled with isopropanol (*V*_1_). Samples were left for 5 min, and after that the total volume of isopropanol and isopropanol-impregnated sample level (*V*_2_) was read. Subsequently, materials were carefully removed from the cylinder. The residual isopropyl alcohol volume (*V*_3_) was then recorded. Measurements of the matrices were performed in triplicate. Equations 3 and 4 were used to calculate the porosity and the density of samples, respectively
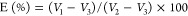
3
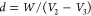
4

#### 2.3.6 Residual Moisture Content

The residual moisture contents of weighed matrices (1 × 1 cm) (*W*_b_) were evaluated as the weight loss of samples dried at 105 °C for 24 h to a constant weight. Subsequently, dried samples were weighed again (*W*_a_). The measurements were carried out in triplicate. The residual moisture contents, defined as the percentage of the water removed from the samples, were calculated as follows (eq 5)
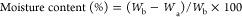
5

### 2.4 Statistical Analysis

In order to statistically compare results, one-way ANOVA with Tukey’s pairwise was performed using the Past 4.09 program (PAleontological Statistics Software, Oslo, Norway). Data are shown as the mean ± standard deviation for each experiment. *p*-Values ≤ 0.05 were considered significant.

## 3 Results and Discussion

### 3.1 Structure of Materials

**Figure 2:**
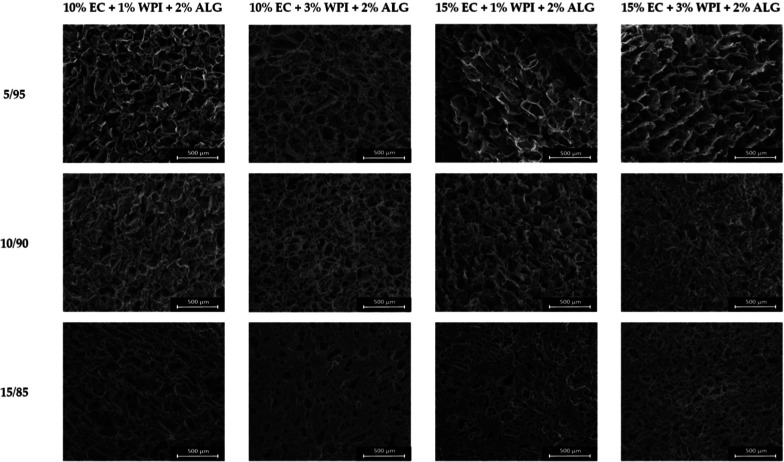
Structure of the obtained freeze–dried bigels in magnification ×150 (scale bar = 500 μm).

**Figure 3:**
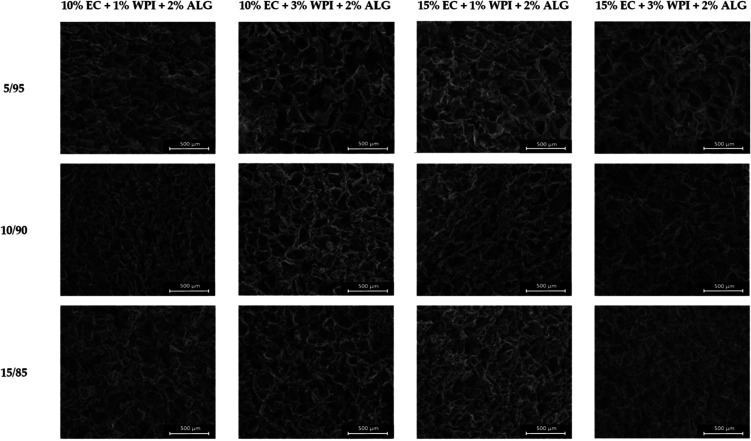
Cross-section of the obtained freeze–dried bigels in magnification ×150 (scale bar = 500 μm).

Freeze–drying of the prepared bigels resulted in obtaining three-dimensional matrices that were soft and spongy. Their structure and cross sections are presented in Figures 2 and 3, respectively. These materials had complex, porous structures with irregular, interconnected macropores. Freeze–dried emulsions maintained a robust porous matrix with an intact structural integrity. Moreover, these materials did not exhibit phase-separated regions. However, an evident difference between samples containing more oleogels was observed. In samples with less oleogel content, the pore walls displayed a rough and wrinkled texture, whereas in materials prepared with 15/85, the pore inner walls were more smooth. The porous network was interspersed with ice crystal imprints, forming a more honeycomb-like structure in samples prepared by using a 5/95 oleogel/hydrogel mixing ratio. Meanwhile, numerous droplet imprints were visible in freeze–dried emulsions containing a 15/85 mixing ratio, suggesting retention of the original emulsion structure. It has been found that the nucleation of ice strongly affects the pore formation.^42^ Since each pore results from the growth of one to a few ice grains within the polymer network, the ice grains are replaced by macropores during the sublimation. Therefore, the resulting structure of the materials is highly porous. The freeze–dried emulsion with higher WPI content exhibited a more compact, uniform, and homogeneous porous structure. Meanwhile, an increased EC concentration resulted in stable, well-formed pores. In lower oleogel/hydrogel mixing ratios, pore walls were wrinkled and folded, giving the structure a rough and irregular texture.

The synthesis process of freeze–dried bigels has a relatively low cost due to the use of widely available and biodegradable raw materials, such as sodium alginate, WPI, glycerin, sunflower oil, and EC. The primary contributors to energy use are heating and freeze–drying. While the environmental impact is low, optimizing freeze–drying efficiency and sourcing renewable energy could further enhance sustainability.

The long-term stability of freeze–dried bigel-based hybrid materials is high due to the strong interactions between the hydrogel and oleogel phases, forming a robust and cohesive network. WPI, sodium alginate, and EC provide structural reinforcement, while freeze–drying significantly enhances durability by reducing water content and preventing microbial growth.

Reports regarding freeze–dried bigels are limited. Martín-Illana et al. focused on formulating these materials with vaginally controlled release of tenofovir. Freeze–dried bigels containing pectin, chitosan, or hypromellose^38^ and guar gum hydrogel and sesame oil containing Span 60 or Span 60 and Tween 60 as surfactants^39^ had porous structures. They also noticed that the microstructure of the materials depended on the type of polymer used and their concentrations. Smaller pores were observed in samples with a larger amount of polymers. This can be ascribed to the greater viscosity and denser polymeric framework produced, resulting in smaller water droplets being trapped inside the network that were sublimated during freeze–drying, thus creating smaller pores.

### 3.2 Swelling Properties

**Figure 4:**
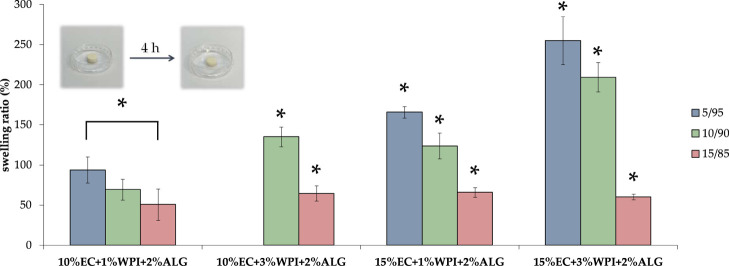
Swelling ratio of freeze–dried bigels. The pictures present an exemplary sample (15% EC + 1% WPI + 2% ALG) before and after 4 h of incubation in PBS buffer. ANOVA-one way with Tukey’s pairwise (Cl = 95%) was performed to compare the results statistically. Significant differences among one group of materials with different oleogel/hydrogel mixing ratios were marked on the graph with (*).

Swelling properties were determined by immersing samples in PBS at pH 5.7 for 4 h (Figure 4). The swelling ratio of all porous materials increased due to the solvent uptake from the surrounding medium. The resulting overall swelling ratio differed from 50 to 255%. One sample (10% EC + 3% WPI + 2% ALG_5/95) dissolved. Samples containing 10% EC and 1% WPI + 2% ALG presented the lowest swelling properties in the 50 – 100% range. Increasing the WPI content to 3% resulted in higher swelling ratio (65 – 135%). Furthermore, samples containing a higher amount of EC in oleogel, i.e., 15% and 1% of WPI with 2% of sodium alginate developed swelling measurement values (65 – 165%) similar to 10% EC + 3% WPI + 2% ALG, but higher than 10% EC + 1% WPI + 2% ALG. The highest swelling properties had materials containing the highest amount of EC and WPI, ranging from 60 to 255%. Therefore, increasing the concentrations of WPI and EC in samples with 5/95 and 10/90 oleogel-to-hydrogel mixing ratios resulted in an increase in swelling ratios. Bigels with 15/85 oleogel/hydrogel proportions did not show significant differences in terms of polymer concentrations. Meanwhile, increasing oleogel content in the material composition led to a decrease in samples’ swelling properties. The lowest swelling properties of samples containing the highest content of oleogel may be attributed to their lower disintegration due to the higher amount of oil in the material composition. Based on the obtained results, one can conclude that the swelling ratio significantly depended on the polymers content and the oleogel/hydrogel mixing ratio. It was higher when EC and WPI concentrations were higher and the oleogel/hydrogel mixing ratio was lower. Materials with a higher concentration of polymers and a lower proportion of oleogel were able to uptake more swelling medium, resulting in higher swelling properties. A higher amount of polymers with hydrophilic groups increased sponges’ hydrophilicity and enhanced the absorption of water molecules, increasing their swelling properties.^43^

Other studies supported our findings. Alginate bigel-based beads also exhibited a lower swelling rate with the increase in the oleogel content.^44^ Freeze–dried bigels prepared by Martín-Illana et al. based on guar gum hydrogel and sesame oil, adding Span60 or Span60 and Tween60 as surfactants, had a maximum swelling degree from 60 to 260%.^39^ They also noticed that a smaller amount of polymers and a higher oil content led to a lower swelling ratio. This may be ascribed to the more consistent microstructure of the materials, allowing them to maintain their structure for extended periods. Manzocco et al. obtained WPI materials using freeze–drying and supercritical drying and characterized them by water uptake capacity.^45^ Despite the difference in these samples’ morphology, porosity, and kinetics of water absorption in time, their swelling ratio was approximately 45 – 50%. Nonetheless, the water uptake led to the destruction of materials. Sodium alginate is a component of numerous freeze–dried materials. Gelatin/alginate sponges have been reported to have a swelling ratio in the range between 100 to 900%, depending on the time of immersion and the proportions of polymers.^46^ Higher swelling properties and faster degradation of samples comprising a greater amount of alginate may be linked to larger pores in sponge-like materials, which boosted the swelling rate. However, the swelling capacity of chitosan/alginate/hyaluronic acid materials was revealed to have values from 75 to 175%, with higher water uptake ability of samples containing more alginate.^47^ Therefore, several parameters impact the swelling properties of porous materials, such as porosity, the hydrophilicity of polymers, the structure of polymer networks, and the interactions between polymer chains.^48^

### 3.3 Degradation Properties

Freeze–dried polymeric bigels were degraded in PBS (pH 5.7) for 7 days. Ours freeze–dried bigel-based materials were fabricated from degradable polymers: WPI, sodium alginate, and EC. Samples were not cross-linked since we designed these materials to be easily dissolved back to bigels immediately before their topical use on the skin as a skin-conditioning product. Therefore, degradation measurements were carried out in pH corresponding to the skin’s natural pH.

**Figure 5:**
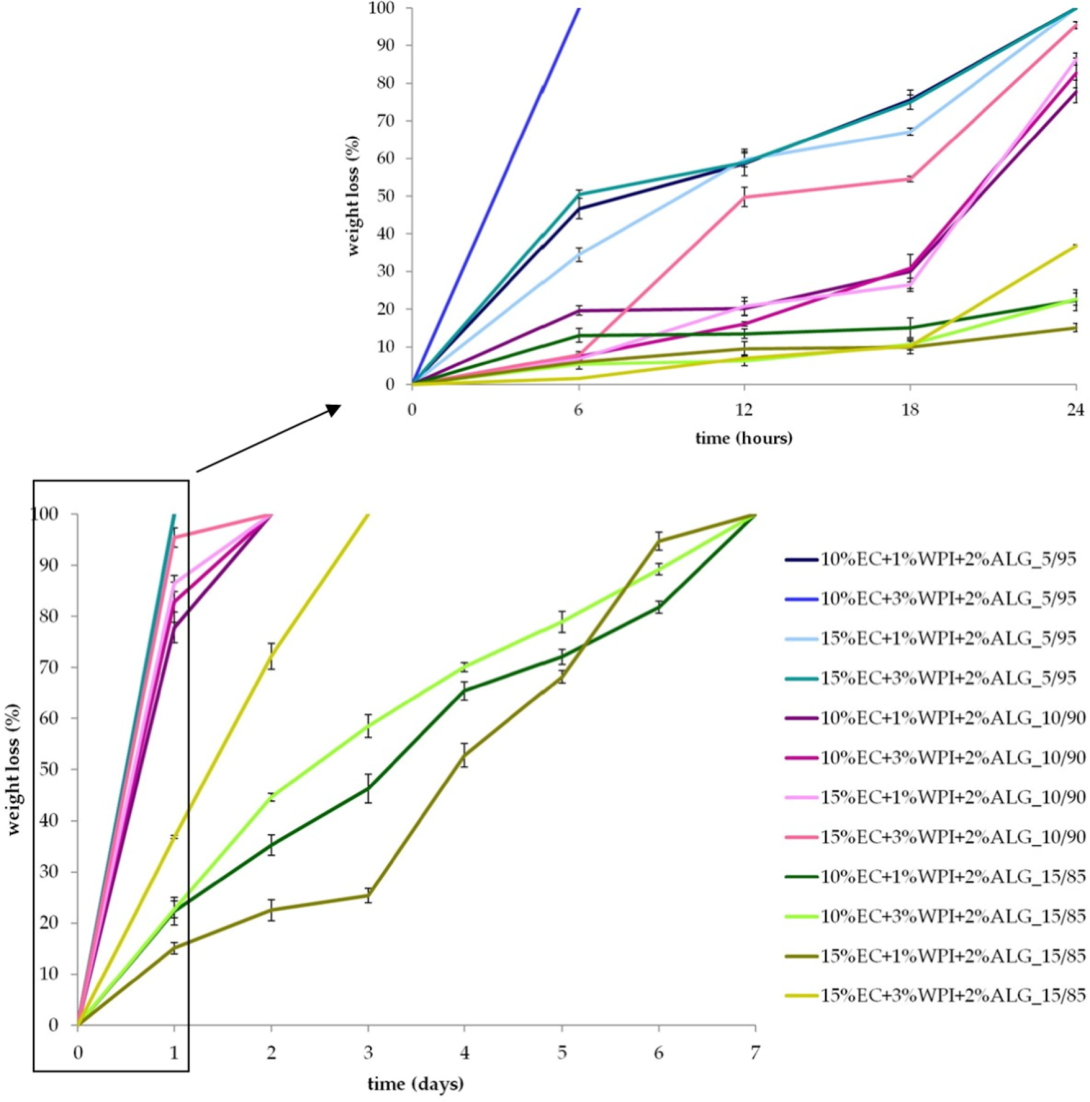
Values of weight loss during degradation measurements of freeze–dried bigels.

According to the results (Figure 5), the percentage weight loss strongly depended on the hydrogel/oleogel mixing ratio. Therefore, samples containing less oleogel phase (5/95) degraded within the first 24 h. Freeze–dried bigels obtained with a 10/90 oleogel/hydrogel mixing ratio fully degraded after 2 days. Degradation of materials containing the highest amount of oleogel in their composition occurred within 7 days of analysis. Therefore, an important observation was that introducing a higher contribution of hydrophobic oleogel delayed the degradation of bigels, providing protection from degradation processes. The higher contribution of oleogel in the samples’ composition resulted in a lower degradation rate due to the hydrophobic nature of the oleogel, which reduces water uptake and minimizes susceptibility to hydrolytic degradation. EC in the oleogel forms a stable nonpolar matrix that shields the material from environmental moisture, enhancing its structural integrity over time. This protective effect contrasts with hydrogel-rich compositions, which are more prone to water absorption and subsequent degradation due to their hydrophilic characteristics. However, samples containing higher concentrations of polymers, 15% of EC in oleogel, and 3% of WPI in hydrogel with 10/90 and 15/85 mixing ratios tend to have higher percentage weight loss than other samples. The higher degradation in samples with increased polymer content (15% EC and 3% WPI) is due to the hydrophilic nature of WPI, which enhances swelling and allows for greater medium penetration into the matrix. This increased medium uptake accelerates hydrolytic degradation, particularly in hydrophilic components such as WPI and sodium alginate, weakening the structural stability. Sodium alginate plays a critical role in the structural stability and swelling behavior of bigel-based materials. Its hydrophilic nature promotes water absorption and swelling, enhancing medium penetration into the matrix and potentially accelerating degradation. The degradation of samples is in agreement with the results obtained for the swelling properties. Increased swelling properties can enhance medium penetration into the polymer matrix, accelerating their degradation process.^49^ Hence, the higher the medium uptake abilities, the higher the degradation of the obtained bigel-based materials. This observation is strictly related to their composition (content of polymers) and oleogel/hydrogel mixing ratio.

The degradation rate of freeze–dried polymeric materials depends on several factors, such as their structure and composition, moisture content, and porosity.^50^ It was noted that hydrolysis and proteolysis are essential mechanisms for the degradation of materials based on polysaccharides and proteins. This leads to the depolymerization of polysaccharides into monomers influenced by water molecules, leading to the cleavage of chemical bonds^51^ or the breakdown of proteins into smaller peptides or amino acids^52^ and, eventually, material breakdown. Nonetheless, incorporating lipids into freeze–dried polymeric materials may lead to complex changes in their degradation behavior, influenced by factors such as moisture absorption, chemical interactions, and mechanical properties.^53,54^ The hydrophobic nature of oleogel can reduce the polymer matrix’s overall water absorption, slowing down hydrolytic degradation. Quickly degradable materials are increasingly sought by scientist for various applications due to their environmental benefits and potential for sustainability.^55^ Some critical applications may include packaging, pharmaceuticals, agriculture, and personal care products.

### 3.4 Mechanical Properties

The values of Young’s modulus and compressive and yield strength significantly depended on the content of polymers: WPI in hydrogel and EC in oleogel, as well as their mixing ratio (Table 2). Samples containing 3% of WPI exhibited a rise in the values of Young’s modulus and compressive and yield strengths with an increasing oleogel/hydrogel mixing ratio. However, the mechanical parameters decreased with the increasing oleogel/hydrogel mixing ratio in samples containing 1% of WPI. Young’s modulus decreased with a higher oleogel-to-hydrogel mixing ratio since the oleogel phase was softer and less rigid than the hydrogel phase. As the oleogel content increased, it disrupted the denser and more interconnected network provided by the hydrogel, reducing the material’s overall stiffness. This shift in composition led to a more compliant structure, resulting in lower resistance to deformation under stress and, consequently, a lower Young’s modulus. The results indicated that samples containing 1% WPI, 2% sodium alginate, and 15% EC in a 5/95 oleogel/hydrogel mixing ratio (∼3.7 MPa) were the stiffest materials showing the most resistance to compression.

**Table 2:** Mechanical Properties of the Obtained Freeze–Dried Bigels Based on WPI, Sodium Alginate (ALG), and Ethylcellulose (EC) during Compression

sample	Young’s modulus (MPa)	compressive maximum force (N)	yield strength (N/mm^2^)
10% EC + 1% WPI + 2% ALG_5/95	2.46 ± 0.45	15.5 ± 0.49	8.03 ± 0.27
10% EC + 1% WPI + 2% ALG_10/90	2.21 ± 0.62	12.7 ± 0.42	5.69 ± 0.65
10% EC + 1% WPI + 2% ALG_15/85	1.91 ± 0.64	9.70 ± 0.37	3.59 ± 0.27
10% EC + 3% WPI + 2% ALG _5/95	1.25 ± 0.22	4.36 ± 0.40	1.83 ± 0.48
10% EC + 3% WPI + 2% ALG _10/90	1.31 ± 0.44	5.12 ± 0.34	2.13 ± 0.41
10% EC + 3% WPI + 2% ALG _15/85	2.70 ± 0.46	5.54 ± 0.24	2.22 ± 0.52
15% EC + 1% WPI + 2% ALG_5/95	3.73 ± 0.77	12.7 ± 0.37	7.26 ± 0.37
15% EC + 1% WPI + 2% ALG_10/90	2.32 ± 0.90	12.0 ± 0.20	6.46 ± 0.40
15% EC + 1% WPI + 2% ALG_15/85	1.78 ± 0.22	9.82 ± 0.47	5.52 ± 0.14
15% EC + 3% WPI + 2% ALG_5/95	1.29 ± 0.47	5.71 ± 0.08	3.07 ± 0.19
15% EC + 3% WPI + 2% ALG_10/90	1.43 ± 0.45	5.78 ± 0.08	2.65 ± 0.32
15% EC + 3% WPI + 2% ALG_15/85	1.94 ± 0.20	5.84 ± 0.17	3.24 ± 0.29

Bigel-based beads containing alginate hydrogel and glycerol monostearate oleogel exhibited a decrease in Young’s modulus and hardness with the increase in oleogel contribution.^44^ However, increasing the oleogel content led to an increase in hardness (from ∼ 0.1 N to ∼ 2 N) of gelatin-glycerol monostearate bigels with the best mechanical properties for samples homogenized for 3 min.^56^ Freeze–dried bigels based on organogel (sesame oil and Span 60) and hydrogel (pectin, chitosan or HPMC and Tween60) were increasingly deformable, and they presented hardness from 1.21 to 6.48 N.^38^ Aerogel prepared from an oil-in-water emulsion containing WPI and soybean oil modified by the addition of guanidinium hydrochloride presented high yield stress (1.4 MPa) and Young’s modulus (16.9 MPa).^57^ The authors linked it to the cellular structure and the synergistic effect of enhanced intermolecular disulfide bonds and oil droplets working as cross-linkers. Chen et al. obtained foam-like materials based on WPI and blends of WPI with alginate in order to improve their mechanical properties during compression.^58^ It revealed that pure WPI aerogels were very brittle (0.18–1.6 MPa) depending on the concentration of WPI. However, the greater addition of alginate resulted in further modulus improvement from 0.48 to 12.9 MPa. Alginate/gelatin materials had a maximum elastic stress of around 0.4 and 0.2 MPa and Young’s modulus of approximately 3.5–4 MPa,^59^ 6.7 MPa, and after additional modification with usnic acid 2.3 and 21.1 MPa.^60^ However, porous materials based on alginate blended with different polymers have not always been shown to exhibit enhanced mechanical properties. Adding alginate to bacterial cellulose sponges decreased Young’s modulus from 3 MPa to less than 1 MPa.^61^ In comparison, alginate/chitosan sponges were noticed to have a less defined microstructure than the single component sponges, resulting from a polymeric network being more randomly ordered during the freezing of samples.^62^ The compression force of chitosan samples was ∼5.5 N and that of alginate materials was less than 0.5 N, while their mixtures had compression force in between those results.

### 3.5 Porosity and Density Measurements

**Figure 6:**
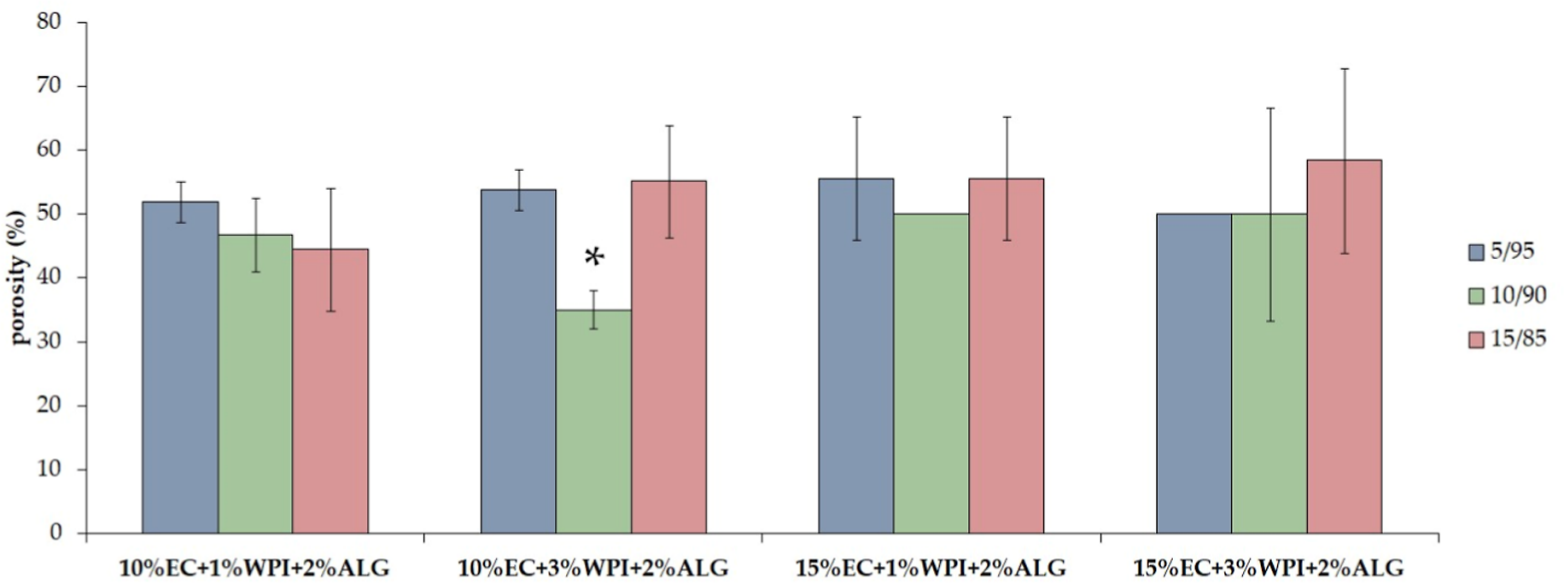
Porosity of freeze–dried bigels based on WPI, sodium alginate, and ethylcellulose (EC). ANOVA-one way with Tukey’s pairwise (Cl = 95%) was performed to statistically compare the results. Significant differences among one group of materials with different oleogel/hydrogel mixing ratios were marked on the graph with (*).

**Figure 7:**
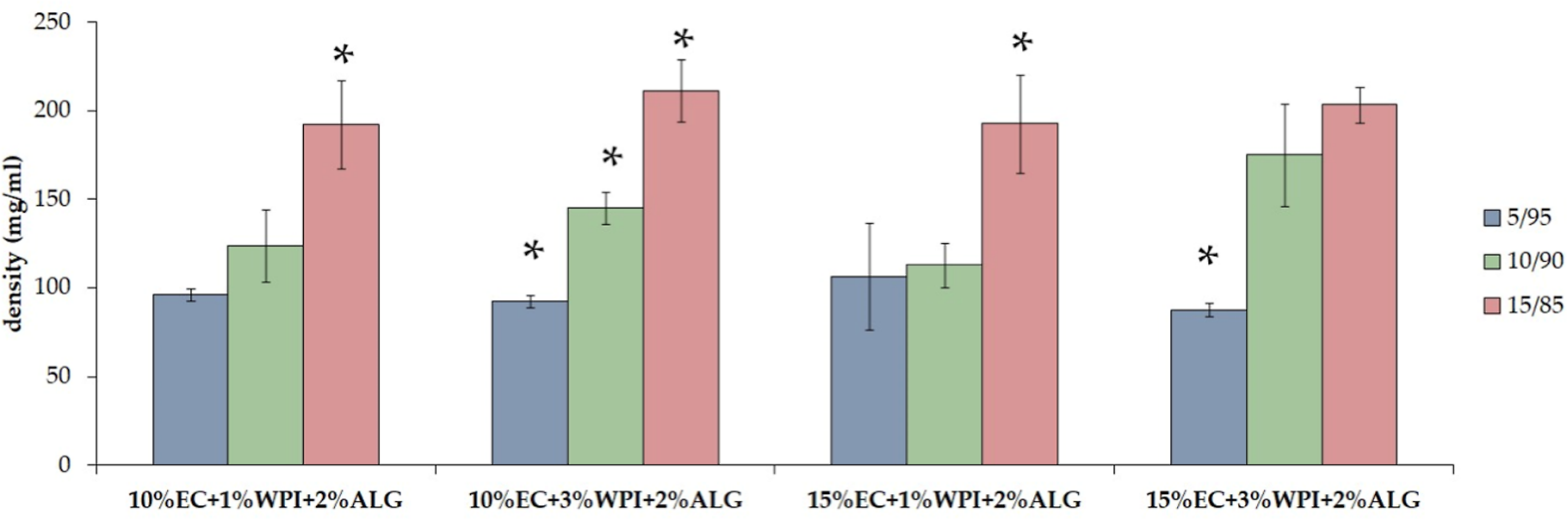
Density of freeze–dried bigels based on WPI, sodium alginate, and EC. ANOVA-one way with Tukey’s pairwise (CI = 95%) was performed to statistically compare the results. Significant differences among one group of materials with different oleogel/hydrogel mixing ratios were marked on the graph with (*).

Porosity and density were evaluated via liquid displacement using isopropyl alcohol (Figures 6 and 7). Porosity showed no significant differences in terms of different polymer contents and the oleogel/hydrogel mixing ratio. The porosity of freeze–dried bigels based on EC/sunflower oil oleogel and WPI/alginate hydrogel ranged from 45 to 58%. The sample 10% EC + 3% WPI + 2% ALG_10/90 was an exception, which showed a significantly lower porosity (35%).

The formation of pores is strictly related to the nucleation of ice during the freezing of materials and the sublimation of ice crystals during the freeze–drying process.^42^ Porosity and the shape and size of pores are linked to the morphology of ice crystals. Hence, these parameters can be adjusted depending on the purpose of the application of the materials. Freeze–dried materials based on alginate alone or in combination with different polymers, such as chitosan and gelatin, have been reported to have a wide range of porosity: 38–57%,^63^ 30–90%,^64^ ∼ 90%,^65^ and 92%.^66^

Based on the measurements (Figure 7), the density of freeze–dried bigels showed a difference from around 100 mg/mL (for samples with a 5/95 oleogel/hydrogel mixing ratio) to 200 mg/mL (for materials containing oleogel/hydrogel mixing ratio: 15/85). This indicates that this parameter increased with the increase of the oleogel ratio in materials. However, we did not observe differences in the density of samples containing different amounts of polymers: WPI in hydrogel or EC in oleogel.

Although xanthan and guar gum-based bigels displayed significantly higher densities (790–840 mg/mL^67^), the results of the materials after freeze–drying aligned with ours. Manzocco et al. fabricated WPI aerogels with low density ranging from 0.22 to 0.29 g/cm^3^, which depended on the preparation method: freeze–drying of samples resulted in lower-density materials than supercritical–CO_2_–drying.^45^ However, Chen et al. observed differences in the density of WPI/alginate materials depending on the polymers contents. WPI-based porous materials exhibited density ranging from 0.112 to 0.245 g/cm^3^, increasing with the higher content of WPI, whereas foam-like material based on alginate had a density of 0.047 g/cm^3^.^58^ They also prepared samples blending WPI and alginate with a density differing from 0.0592 to 0.129 g/cm^3^, indicating that the addition of alginate decreased the density of the materials. Materials based on alginate blended with different components showed similar densities. Foams based on alginate, potato starch, and the clay mineral sepiolite prepared by Darder et al. presented density from 0.123 to 0.242 g/cm^3^ with higher density for samples cross-linked with calcium ions.^68^ The density of alginate/halloysite nanotube composite scaffolds ranged from 0.035 to 0.139 g/cm^3^, with the lowest density for pure alginate samples.^69^ The researchers explained the increased density of composite materials to the greater constituent content in the material volume since the water volume was adjusted during the preparation of samples.

### 3.6 Residual Moisture Content

**Figure 8:**
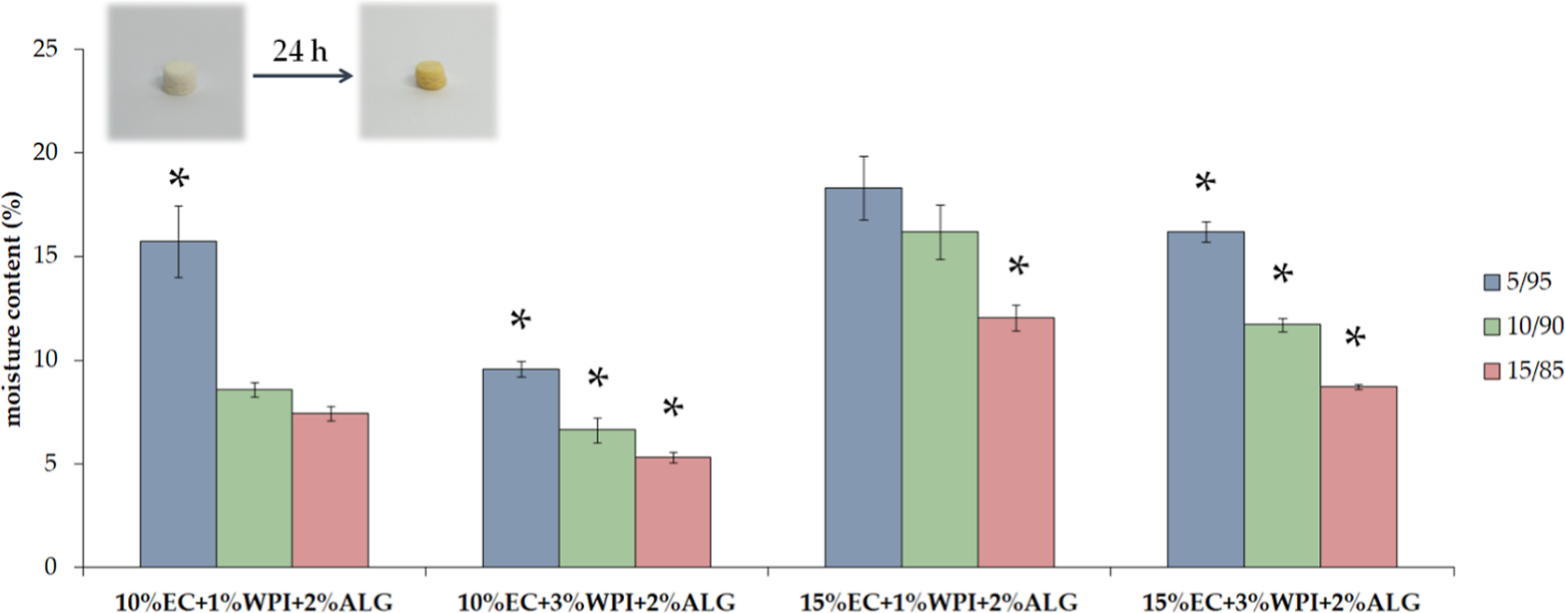
Residual moisture content of freeze–dried bigels. The pictures present an exemplary sample (15% EC + 1% WPI + 2% ALG) before and after 24 h of drying at 105 °C. ANOVA-one way with Tukey’s pairwise (Cl = 95%) was performed to statistically compare the results. Significant differences among one group of materials with different oleogel/hydrogel mixing ratios were marked on the graph with (*).

The residual moisture content analysis was performed by drying the samples at 105 °C for 24 h (Figure 8). It is a crucial parameter determining the stability of obtained freeze–dried materials. The moisture content of freeze–dried bigels significantly depended on their composition due to their components’ hydrophilic and hydrophobic characteristics, as well as their interactions during the freeze–drying process. The moisture content increased with higher EC content since it created a denser oleogel network that might entrap small amounts of residual water within its matrix. Conversely, increasing the WPI content reduced the moisture content as WPI promoted stronger protein–protein interactions during gel formation, which enhanced water removal during freeze–drying. Similarly, a higher oleogel-to-hydrogel mixing ratio decreases moisture content as the hydrophobic oleogel phase limits water retention. Therefore, the moisture content in these freeze–dried bigels was from 5 to almost 20%.

The results of residual moisture content measurements were in agreement with those of other studies. Freeze–dried hydrogels based on calcium alginate with loaded ciprofloxacin presented moisture content from 13.28% to 17.30%.^70^ Meanwhile, sponges prepared from the alginate and acacia gum mixture cross-linked with CaCl_2_ had moisture content in the 7–27% range with potential as wound dressing.^71^ It was established that the residual moisture content is highly dependent on secondary drying temperatures.^72^ Freeze–drying is a multistep process consisting of freezing the sample and primary drying, which is the sublimation of frozen water under vacuum. The last step is secondary drying, when unfrozen water from samples is removed by desorption at elevated temperatures. An optimal residual moisture content has been demonstrated not to be the lowest possible since very low moisture content accelerates oxidation and degradation of proteins.^73,74^

### 3.7 General Discussion

The exact mechanisms affecting the stability of freeze–dried bigels remain unclear, but they may be dictated by a combination of hydrogen bonding, electrostatic interactions, van der Waals forces, and hydrophobic associations between the hydrogel (WPI/sodium alginate/glycerin) and oleogel (EC/sunflower oil/Span 80) phases. Within the hydrogel, WPI—rich in β-lactoglobulin and α-lactalbumin—may have stabilized the network through protein–protein interactions, including disulfide bridge formation and hydrogen bonding,^75^ as well as protein–polysaccharide interactions, namely electrostatic interactions with sodium alginate.^76^ Meanwhile, sodium alginate not only enhanced structural integrity via electrostatic interactions but also contributed to water retention and swelling due to its highly hydrophilic carboxyl and hydroxyl groups. Furthermore, glycerin, acting as a plasticizer, disrupted excessive protein aggregation and enhanced the flexibility of the materials. The oleogel phase was structured by EC, which may have formed a gel network through hydrogen bonds during thermally induced gelation, with sunflower oil trapped within the polymeric matrix.^77,78^ In addition, Span 80 may have acted as a nonionic surfactant to improve interfacial adhesion between the two immiscible gel phases, preventing phase separation during freeze–drying.

The porosity of these bigels was primarily influenced by the freeze–drying process, where ice crystal formation dictated the pore structure. Higher oleogel content led to a denser, more compact matrix (Figure 7), while a lower oleogel content resulted in an enhanced residual moisture content (Figure 8) and swelling properties (Figure 4). Swelling behavior will have been driven by the hydrophilicity of the polymer network; sodium alginate is highly hydrophilic and molecules in WPI contain hydrophilic regions. The presence of these molecules will have enabled water uptake, increasing swelling capacity. In contrast, the hydrophobic oleogel phase limited water penetration, thereby reducing swelling. The mechanical properties of the bigels, as shown in Table 2, depended on the balance between hydrogel elasticity and oleogel rigidity, with higher EC content increasing stiffness and Young’s modulus. Degradation of the freeze–dried gels might have occurred through hydrogel dissolution and hydrolysis. Although higher WPI content may have increased stability, as discussed above, it may have accelerated breakdown due to increased water absorption and solvent accessibility, while higher EC content and oleogel-rich formulations may have provided a hydrophobic barrier that slowed degradation, extending it to 7 days (Figure 5). It should be noted that this discussion remains speculative; the elucidation of the exact mechanisms remains a topic for further study.

These molecular interactions allow for precise tuning of the bigels’ physicochemical properties, enabling controlled swelling, degradation, and mechanical performance, making them promising candidates for dermatological applications, transdermal drug delivery, and advanced biomaterial formulations with tailored release and enhanced stability.

## 4 Conclusions

In this study, functional freeze–dried bigels based on a WPI/sodium alginate/glycerin hydrogel and EC/Span 80/sunflower oil oleogel were successfully formulated. Physicochemical properties, such as swelling, degradation and mechanical properties, moisture content, and density significantly depended on the content of biopolymers in samples: WPI (1% or 3% in hydrogel) and EC (10% or 15% in oleogel), as well as the oleogel/hydrogel mixing ratio (5/95, 10/90, or 15/85). The lower the oleogel ratio and the higher the EC concentration in prepared samples, the higher their swelling ratio (from ∼50 to ∼ 255%) and moisture content (∼5 to ∼20%). The mechanical properties of freeze–dried bigels described as Young’s modulus, compressive strength, and yield strength ranged from 1.25 to 3.7 MPa, with the highest result for the sample containing 15% of EC in oleogel and 1% of WPI in hydrogel with 5/95 oleogel/hydrogel mixing ratio. The porosity of formulated materials (from 45 to 58%) did not significantly differ in terms of studied variables except for the lowest value (35%) for the sample containing 10% EC in oleogel and 3% WPI in hydrogel with a 10/90 mixing rate. A rise in density (from ∼ 100 to 200 mg/mL) and prolonged degradation (from 6 h to 7 days) with the increase in the oleogel content in bigels was observed.

These findings highlight the potential of freeze–dried bigels as versatile and customizable biomaterials not only for dermatological and cosmetic applications. Considering both structured phases of bigels and resulting from these additional benefits, the possibility of simultaneous addition of hydrophilic and lipophilic active substances, stability, and good characteristics for skin application makes them promising candidates for advanced skincare formulations, wound healing applications, and transdermal drug delivery systems. Furthermore, their tunable swelling and degradation profiles offer opportunities for controlled release applications. Therefore, future work should focus on evaluating the biological compatibility and efficacy of these drugs in delivering active compounds for dermatological and cosmetic use. This involves conducting application tests after hydration, such as rheological evaluation, spreadability assessment, and in vitro analysis of skin biophysical parameters as well as optimizing their composition for enhanced bioactivity and incorporating therapeutic agents for specific applications to further expand their potential.

## Data Availability

The data that support the findings of this study are available from the corresponding author upon request.
